# Neuroimmune Interactions and Rhythmic Regulation of Innate Lymphoid Cells

**DOI:** 10.3389/fnins.2021.657081

**Published:** 2021-04-29

**Authors:** Nicolas Jacquelot, Gabrielle T. Belz, Cyril Seillet

**Affiliations:** ^1^Walter and Eliza Hall Institute of Medical Research, Parkville, VIC, Australia; ^2^Department of Medical Biology, University of Melbourne, Parkville, VIC, Australia; ^3^Diamantina Institute, The University of Queensland, Woolloongabba, QLD, Australia

**Keywords:** circadian rhythm, neuroimmune interactions, homeostasis, inflammation, neuropeptide

## Abstract

The Earth’s rotation around its axis, is one of the parameters that never changed since life emerged. Therefore, most of the organisms from the cyanobacteria to humans have conserved natural oscillations to regulate their physiology. These daily oscillations define the circadian rhythms that set the biological clock for almost all physiological processes of an organism. They allow the organisms to anticipate and respond behaviorally and physiologically to changes imposed by the day/night cycle. As other physiological systems, the immune system is also regulated by circadian rhythms and while diurnal variation in host immune responses to lethal infection have been observed for many decades, the underlying mechanisms that affect immune function and health have only just started to emerge. These oscillations are generated by the central clock in our brain, but neuroendocrine signals allow the synchronization of the clocks in peripheral tissues. In this review, we discuss how the neuroimmune interactions create a rhythmic activity of the innate lymphoid cells. We highlight how the disruption of these rhythmic regulations of immune cells can disturb homeostasis and lead to the development of chronic inflammation in murine models.

## Introduction

The innate immune system is often seen as the first line of defense against invading pathogens, but it is now evident that they also carry out homeostatic functions by regulating essential pathways involved in tissue repair, mucosal barrier function, and metabolism. These functions have been particularly highlighted with the discovery of the innate lymphoid cells (ILCs) in early 2010’s (Vivier et al., 2018). In contrast to B and T lymphocytes, ILC activity is not modulated by antigen-specific receptors but by a dynamic integration of host-derived physiological signals (Seillet and Jacquelot, 2019). The ILC family comprises NK cells, ILC1, ILC2, and ILC3. Their constitutive activity at the body’s barrier surfaces ensures the maintenance of tissue homeostasis and immune protection (Vivier et al., 2018). ILC1 and NK cells are mainly involved in responses against intracellular pathogens and tumor surveillance (Seillet et al., 2020a). ILC2 initiate type-2 immune responses which are critical to allergy and anti-parasite responses (Fallon et al., 2006). They also mediate tissue repair through the secretion of amphiregulin (Monticelli et al., 2011). Enhanced ILC2 function in the lung is associated with asthma (Chang et al., 2011) while in adipose tissue, decreased ILC2 cytokine production is associated with obesity and insulin resistance (Molofsky et al., 2013). ILC3 are greatly enriched in the intestine where they mediate anti-bacterial responses (Sawa et al., 2011). They produce the interleukin (IL)-22 which promotes colonization of the gut by beneficial commensal bacteria that protect against intestinal inflammation (Pickard et al., 2014). Decreased ILC3 functions are associated with impaired capacity to maintain the mucosal barrier intact (Satoh-Takayama et al., 2014). Recent studies have shown that ILC responses are modulated by rhythmically expressed neuropeptides. These recent advances could contribute to the understanding of the mechanisms that leads to increased incidence of chronic inflammatory diseases when circadian rhythms are disrupted.

**FIGURE 1:**
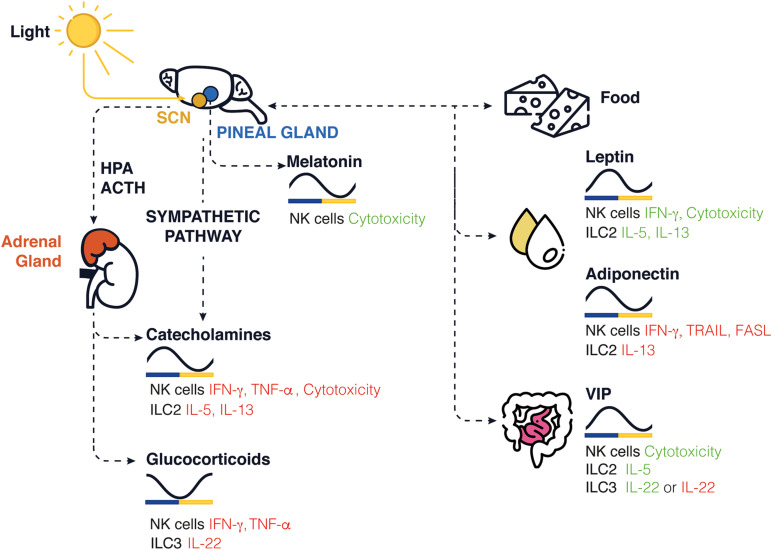
Schematic representation of circadian clock-mediated control of innate immune cells in rodents. In the brain, the SCN controls the rhythmic expression of GCs and catecholamines released in periphery, while the pineal gland controls the release of melatonin. The SCN controls the daily feeding/fasting (activity/rest) cycles, whereas food intake, stress hormone, sleep, and locomotor activity entrains and synchronizes peripheral clocks and the local release of neuropeptides such as leptin, adiponectin, and VIP. The levels found in nocturnal rodents for each molecule are shown across a 24-h period. Green/red represents an increase/decrease in cytokine production or cytotoxicity function.

Circadian rhythms are endogenous oscillations with a period close to 24 h. They are found in almost all living organisms. The temporal alignment of the internal physiology with the external environment is critical for survival and the evolution of species. Circadian rhythms are found in virtually all cells of the body and function autonomously. However, these oscillations need to be synchronized with the environment, and external signals such as the light, temperature, and food intake (Reppert and Weaver, 2002). Therefore, our lifestyle, physical activity, and feeding times are important components for the robustness of these rhythms. Light sensed by the retina is connected to the suprachiasmatic nucleus (SCN) in the hypothalamus. The SCN is also known as the master clock and is responsible for entraining peripheral circadian clocks distributed across the organism. All peripheral clocks are synchronized daily and coordinated by the SCN via the hypothalamic pituitary adrenal (HPA) axis and the autonomic nervous system (ANS; Gnocchi and Bruscalupi, 2017; Figure 1). Food intake can be aligned to natural feeding rhythms induced by the SCN and contribute to synchronize the peripheral clocks. Food intake can also be desynchronized with the SCN due to environmental changes, such as food restriction or temporally altered behavior.

Circadian rhythms are generated in SCN neurons using transcriptional feedback loops that take 24 h to complete. In mammals, the main loop is initiated by the transcription factors CLOCK and BMAL1 (Reppert and Weaver, 2002). These proteins induce the expression of period genes (Per1, Per2, and Per3) and cryptochrome genes (Cry1 and Cry2) that will then inhibit the expression of CLOCK and BMAL1 genes (Xu et al., 2015). This core feedback loop is modulated by additional transcription factors involving Rev-Erbα and the retinoic acid receptor (RAR)-related orphan receptor (ROR) family that ensures the rhythmic expression of BMAL1 (Sato et al., 2004; Yanofsky et al., 2013). Finally, the D-box binding protein (DBP) can activate BMAL1 while Nfil3 will inhibit the expression of clock genes (Mitsui et al., 2001). These transcription factors not only regulate the expression of their own inhibitors but also drive the rhythmic accumulation of target genes also known as clock-controlled genes (Zhang et al., 2014).

In addition to this molecular regulation, the central clock acts as a pacemaker that temporally aligns the peripheral clocks through neural outputs from the ANS, glucocorticoid (GC) hormones and a variety of neuropeptides released by nerves within tissues. While the regulation of ILC by the circadian molecular clock genes have been recently reviewed elsewhere (Wang and Colonna, 2020), we will highlight how the nervous and immune systems interact together. We will discuss the impact of neurohormones on immune cell activity and how they can potentially rhythmically modulate ILC responses.

## Sympathetic Autonomous Nervous System and the Hypothalamic–Pituitary–Adrenal Axis

### Adrenergic System

The adrenergic system is regulated by the sympathetic nervous system (SNS), through the production of catecholamines [epinephrine (EP), norepinephrine (NE)], and via HPA axis. Circulating catecholamine levels exhibit circadian rhythmicity, with EP levels typically rising during the day and falling at night in humans (Linsell et al., 1985) and in an opposite way in rats (De Boer and Van der Gugten, 1987; Schiffer et al., 2001) while data in mice are lacking. EP and NE engage β1- and β2-adrenergic receptors (β2AR), which are commonly found on bronchial smooth and cardiac muscles. Modulating β-adrenergic receptor signaling is, therefore, a common pharmacological strategy used for treatment of asthma (Sears et al., 1990) and cardiovascular conditions (Milano et al., 1994).

The activation of β2AR in human NK cell line inhibits TNF-α, IFN-γ, granzyme B, and perforin expressions (Sun et al., 2018). In humans, EP induces the mobilization of cytotoxic lymphocytes including the NK cells in the circulation (Dimitrov et al., 2010) which could explain the increased number of circulating NK cells during the active period when EP peaks (Dimitrov et al., 2007). In mice, adrenergic signaling inhibits the cytotoxic activity of the NK cells (De Lorenzo et al., 2015), controls NK cell expansion during viral infection (Diaz-Salazar et al., 2020), and inhibits IFN-γ production in hepatic NK cells (Wieduwild et al., 2020). This inhibition was not observed in ILC1 in the liver or splenic NK cells, suggesting a tissue and cell-specific control. In the absence of β2AR signaling, liver NK cells had higher IFN-γ production that resulted in increased resistance to infections associated with better control of viral replication and reduced tissue damage (Wieduwild et al., 2020). A rhythmic expression of TNF-α, IFN-γ, and granzyme B has been observed in NK cells (Logan et al., 2011). Logan et al. reported that levels of NE peaked in the morning in the spleen of rats, and found that the levels of TNF-α, IFN-γ, and granzyme B expression was inhibited during the light phase (resting phase in rodents), while during dark phase, when levels of norepinephrine are reduced, their transcripts were increased (Logan et al., 2011). It is interesting to note that in rats, peak of NE in the spleen is different from the EP level found in the blood, peaking during the active period at night (De Boer and Van der Gugten, 1987; Schiffer et al., 2001). This highlights how neurotransmitters can locally modulate the activity of immune cells. Following splenic sympathectomy, oscillation of granzyme-B, and TNF-α expression in NK cells are abolished, demonstrating the role of the SNS in the entrainment of their rhythmic expression in these cells. This circadian regulation of NK cells by adrenergic signaling could explain the reduced cytotoxicity of NK cells in animals under chronic shift-lag (Logan et al., 2012) and sleep deprivation (De Lorenzo et al., 2015).

Norepinephrine also inhibits type 2 immune responses by impairing ILC2 proliferation and function through its binding to β2AR (Moriyama et al., 2018). After infection with *N*. *brasiliensis*, β2AR-deficient mice have increased intestinal ILC2 infiltration and ILC2-derived IL-13 production. Thus, β2AR acts as a molecular rheostat to control innate immune responses to prevent excessive tissue damage and the development of chronic inflammation (Moriyama et al., 2018). However, a direct link between β2AR signaling and the circadian regulation of ILC2 remains to be established.

### Glucocorticoids

Adrenal glands have a key role in synchronizing peripheral clocks downstream of the SCN through the rhythmic secretion of GC. The GC are steroid hormones and their concentrations in the blood oscillate in a circadian manner, peaking in the morning and nadir at night, in diurnal animals. GC can also be released in pulsatile rhythm. While the circadian expression of GC relies on the SCN and the HPA axis (Spiga et al., 2014), the ultradian rhythm occurs independently of SCN (Waite et al., 2012). GC can regulate the expression of clock genes (Balsalobre et al., 2000). GC-responsive elements are found in Per1/2 genes and Rev-erbα and Rorα are negatively regulated by GC in mice (Surjit et al., 2011).

The GC are well known immunosuppressors of the innate immune responses and are widely used in clinics to treat chronic inflammatory disorders. They have been shown to inhibit the synthesis of various cytokines including TNF-α, IL-1β, IL-2, IL-4, IL-5, IL-6, IL-12, GM-CSF, and IFN-γ in both human and mice (Brattsand and Linden, 1996; Bhattacharyya et al., 2007; Li et al., 2015; Quatrini et al., 2018). GC limits the inflammation and therefore prevents tissue damages. Oscillations in GC concentrations directly impact the rhythmic regulation of the expression of these pro-inflammatory cytokines. Consequently, GC are partially responsible for the observed circadian variations of inflammatory symptoms that suffer patients with asthma and rheumatoid arthritis and many of whom have worsening of symptoms in early morning (Petty, 1988; Cutolo et al., 2003).

In mice, IL-7R expression is regulated by GR in a diurnal rhythm manner and has been shown to promote T lymphocyte survival and recruitment in lymph nodes (Shimba et al., 2018). ILC are strongly dependent on IL-7R signaling for their development and survival but it is still unclear whether GC similarly regulate IL-7R signaling on ILC in a circadian manner. In NK cells, GR deficiency promotes IFN-γ production, reducing mouse survival in response to toxoplasma and mouse cytomegalovirus infections (Quatrini et al., 2018). Similarly, GCs inhibit murine and human ILC3 function as reduced IL-22 production was observed when ILC3 were stimulated with the steroid hormones (Seshadri et al., 2018).

Interestingly, in patients with adrenal insufficiency who require lifelong GC replacement, the circadian administration of GC can partially restore the number of NK cells in circulation (Venneri et al., 2018), while acute administration of cortisol has no effect (Olnes et al., 2016). This suggesting that the timing of GC expression is important to regulate NK cell trafficking.

### Melatonin

At night, the SCN acts on the pineal gland to induce the synthesis of melatonin. Consequently, high plasmatic levels are found in the middle of the night and minimal during the day in mice and humans. Melatonin production is not restricted to the pineal gland, but can also be secreted by the retina, kidneys and the digestive tract in humans (Messner et al., 2001), however, most of the mouse strains do not produce significant amounts of melatonin (Kennaway, 2019). We therefore need to be careful when translating mouse studies to humans. This neurohormone mediates its effects through specific membrane receptors, named melatonin-1 receptors (MT1), MT2, and MT3. In the absence of the pineal gland, the murine NK cell cytotoxic function is decreased. Surprisingly, a single injection of melatonin is able to restore NK cell activity but not when melatonin is administered chronically for 9 days (del Gobbo et al., 1989). Studies have shown a time-dependent influence of melatonin as *in vivo* administration of melatonin induces a significant increase of murine NK cells in spleen and bone marrow (Currier et al., 2000). In pinealectomized rats, the frequency of NK cells in the blood and spleen is increased during the day compared to sham controls (McNulty et al., 1990). Human peripheral lymphocytes cultured in the presence of melatonin show an inhibition of NK cell activity (Lewinski et al., 1989), but chronic administration of melatonin augmented the cytolytic activity and the circulating number of NK cells (Angeli et al., 1988).

## Neuropeptidergic Pathways

### Vasoactive Intestinal Peptide

The vasoactive intestinal peptide (VIP) is a neurotransmitter expressed in neurons found in brain and peripheral tissues such as the lung and gut. In the SCN, VIP is essential for the normal circadian rhythmicity in clock neurons (Aton et al., 2005; Todd et al., 2020) and can induce the expression of PER1/2 (Hamnett et al., 2019). In tissues, VIP is a potent vasodilator but is also involved in other physiological processes, including coordination of gastrointestinal motility, mucus, and enzymatic secretions in response to feeding (Aton et al., 2005; Todd et al., 2020). In mice, food intake induces the release of VIP from enteric neurons creating a rhythmic expression of VIP in the gut (Talbot et al., 2020; Seillet et al., 2020b). The release of VIP in gut and lungs stimulates ILC2 through VIP receptor type 2 (VIPR2) to induce IL-5 production (Nussbaum et al., 2013). The cyclic release of VIP in response to feeding induces a rhythmic production of IL-5 by ILC2s. This circadian expression of IL-5 is detectable in the blood circulation and appears to regulate systemic eosinophil numbers (Nussbaum et al., 2013). In the intestine, VIP-VIPR2 signaling regulates ILC3-derived IL-22 expression (Talbot et al., 2020; Seillet et al., 2020b). While we observed a positive effect of VIP on IL-22 secretion (Seillet et al., 2020b), Talbot et al. (2020) made opposite observations. Despite these, yet unresolved, discrepancies, both studies demonstrated the importance of circadian regulations for ILC function to maintain intestinal homeostasis. While we showed that VIP signaling protects against exacerbated gut inflammation, Talbot and colleagues found that the inhibition of IL-22 by VIP in ILC3 allows the optimal absorption of nutrients (Talbot et al., 2020).

Additional studies have revealed that VIP increased human NK cell cytotoxic function after viral infection (Azzari et al., 1992) and polarized T cell responses by regulating dendritic cells functions. Collectively, these data suggest that VIP can differentially promote inflammation or its resolution in a circadian dependent manner by promoting anti-inflammatory type 2 immune responses, and preventing Th1 infiltration in inflammatory sites.

### Adiponectin

The adiponectin is exclusively secreted by adipocytes, and regulates body energy homeostasis, lipid storage, and adipogenesis (Stern et al., 2016). Its expression is controlled by the clock machinery and peaks at the onset of the feeding phase in mice (Barnea et al., 2015) or early/late morning for human (Gomez-Abellan et al., 2010). Adiponectin induces hypothalamic and peripheral clock genes expression and enhances food intake (Hashinaga et al., 2013; Tsang et al., 2020) as adiponectin-deficient mice have reduced *Bmal1* and *Per2* expressions and reduce food intake during the dark phase (active phase) but experience increase food intake during light phase (resting phase; Tsang et al., 2020). In contrast, overexpression of the adipokine in the liver induces local expression of the clock genes *Bmal1*, *Dbp*, *Cry2*, and *Per2* (Hashinaga et al., 2013), indicating a direct effect of this adipokine on the cell-intrinsic circadian rhythm.

The adiponectin differentially impacts immune cells and can trigger both pro- and anti-inflammatory responses (Luo and Liu, 2016). High levels of adiponectin in mice result in an alteration of the adipose tissue immune cell composition, and a shift operates from a pro- to an anti-inflammatory immune profile leading to an improvement of insulin resistance in models of type 2 diabetes (Kim et al., 2007). NK cells also play a critical role in murine adipose tissue homeostasis, fine-tuning macrophage functions, and dysregulated NK cells are found in obesity (O’Shea and Hogan, 2019; Ferno et al., 2020). Both murine and human NK cells express the adiponectin receptors (Wilk et al., 2013; Jasinski-Bergner et al., 2017). Human NK cells stimulated with various TLR ligands and treated with adiponectin showed reduced IFN-γ production and degranulation capacities (Wilk et al., 2013). Conversely, in adiponectin-deficient mice, while an accumulation of mature NK cells (CD27^*low*^CD11b^*hi*^) is found in the spleen, impaired NK cell degranulation and cytotoxicity are observed and are associated with decreased expression of the activating ligand NKG2D (Wilk et al., 2013). The addition of adiponectin to IL-2 stimulated NK cells leads to impaired cytotoxicity associated with reduced surface expression of FasL and TRAIL, and IFN-γ production (Kim et al., 2006). Recently, adiponectin was shown to suppress ILC2 proliferation and cytokine production and to decrease IL-33-driven ILC2 activation, thus acting as a negative regulator of ILC2 function in adipose tissue (Wang et al., 2021). While no direct link has demonstrated an influence of adiponectin on ILC function in a circadian manner, indirect evidence would suggest a rhythmically regulation of NK cell function by this adipokine. Indeed, adiponectin controls the expression of clock genes which are known to directly influence NK cell activity in rodents (Arjona and Sarkar, 2005, 2006, 2008). The disruption of the *Per2* or *Bmal1* in NK cells reduced IFN-γ, TNF-α, granzyme B, and perforin expressions (Liu et al., 2006; Arjona and Sarkar, 2008). Furthermore, murine NK cell cytotoxic function peaks during the active phase (Arjona et al., 2004; Arjona and Sarkar, 2005, 2006) which coincides with feeding time and higher levels of adiponectin (Barnea et al., 2015; Tsang et al., 2020), however, this interplay remains to be confirmed.

Further studies are warranted to ascertain the role and function of adiponectin on ILC subsets at steady state, over the course of metabolic syndromes, and during circadian misalignment.

### Leptin

Leptin is mainly secreted by adipocytes and follows diurnal variations. In both humans and rodents, the leptin plasma levels peak at night before progressively decreasing, reaching a nadir during the day (Langendonk et al., 1998; Gavrila et al., 2003; Bodosi et al., 2004; Arble et al., 2011). In rodents, starvation induced a decrease in leptin levels and timed-restricted food availability inverted leptin plasma concentrations (Ahima et al., 1996; Bodosi et al., 2004). Furthermore, in humans, circadian misalignment decreases the leptin plasma levels compared to normal alignment (Scheer et al., 2009). Circadian disruptions through thermal lesions of the hypothalamic SCN or in *Cry*^–/–^ and *Per^–/–^* deficient mice completely abolish the diurnal variation of leptin plasma levels (Kalsbeek et al., 2001; Kettner et al., 2015), indicating that circadian clocks control the rhythmic oscillation of the leptin plasma, independently of external food cues, potentially through direct regulation of gene expression. Indeed, the heterodimer BMAL1:CLOCK is capable of binding to the promoter of the leptin gene following a circadian rhythm and regulating C/EBP-α mediated *leptin* transcription (Kettner et al., 2015). Finally, chronic circadian disruption promotes leptin resistance in murine CNS (Kettner et al., 2015) which is known to be associated with obesity (Flier, 2004).

Mice lacking leptin (*ob*/*ob*) or its receptor (*db/db*) expression show immune deficiencies suggesting a direct role of the leptin signaling on the immune system (Bennett et al., 1996; Lord et al., 1998; Howard et al., 1999; Sanchez-Margalet et al., 2003; De Rosa et al., 2007). Both murine and human NK cells express variable levels of the short and long forms of the leptin receptor (Zhao et al., 2003; Lamas et al., 2013; Laue et al., 2015; Keustermans et al., 2017; Bahr et al., 2018). Particularly, obese patients who experience high leptin plasma levels have impaired NK cell phenotype and function, a reversible compromised state when there is a diminution of leptin plasma levels-associated with fat mass reduction (Jahn et al., 2015; Laue et al., 2015; Bahr et al., 2018). In leptin receptor deficient *db*/*db* mice, NK cell numbers in blood, spleen, liver and lungs are all reduced and cells have impaired cytolytic capacities compared to wild type control animals (Tian et al., 2002). In addition, leptin signaling-deficient mice injected with B16 melanoma or LLC cells have increased number of lung metastases compared to control mice (Mori et al., 2006). Stimulation of human NK cells with leptin promotes NK cell metabolism, proliferation, and cytotoxic functions (Zhao et al., 2003; Wrann et al., 2012; Lamas et al., 2013). Interestingly, while short term exposure of NK cells to physiological doses of leptin stimulates NK cells, long-term stimulation inhibits NK cell cytotoxicity and cytokine production (Wrann et al., 2012). Thus, dependent on the levels and duration of the stimulation, leptin may differently influence immune cell responses. The impact of leptin on other ILC subsets is only beginning to emerge. Leptin enhances type-2 responses in allergic rhinitis and stimulates ILC2 proliferation and IL-13 production in both humans and mice (Zheng et al., 2016; Wen et al., 2020; Zeng et al., 2020). Given the role of ILC2 on adipose tissue homeostasis and allergy reactions, leptin and other adiponectin would certainly modulate widely the function of these innate immune cells in health and diseases. Thus, further studies are warranted to delineate the role of these hormones on non-NK cell ILC.

## Concluding Remarks

In our modern societies, the prevalence of circadian rhythm disruption is rising due to our lifestyle or might be imposed by extending working hours and night shift. Nonetheless, circadian rhythm disruption is yet to be recognized as a major public health issue; but accumulating evidence shows that circadian rhythms are an important part of our healing process and control inflammation. As medicine is evolving toward a more personalized approach, circadian regulation of immune responses will be an aspect to consider. Studying circadian rhythms at a global physiological lens will help us to understand how alteration of our natural rhythms by environmental cues or our behavior will impact the metabolism, hormonal signaling and immune function. It is crucial to increase our understanding of the circadian patterns of immune responses and how they are regulated by central and peripheral clocks to enable discovery of chronotherapeutic approaches for optimal timing of therapy administration toward effective measures for treating inflammatory diseases, allergies, and infections. Because animal studies are used to reduce the complexity of parameters influencing circadian rhythms (e.g., temperature, food availability, and external stressor), it will be important to optimize the experimental setups, including the control of external circadian rhythm perturbations (such as, manipulation of light-dark cycles, sleep restriction, or time-restricted feeding) to develop the best translational approaches.

## Author Contributions

All authors listed have made a substantial, direct and intellectual contribution to the work, and approved it for publication.

## Conflict of Interest

The authors declare that the research was conducted in the absence of any commercial or financial relationships that could be construed as a potential conflict of interest.
